# Isolation and Functional Gene Analyses of Aromatic-Hydrocarbon-Degrading Bacteria from a Polychlorinated-Dioxin-Dechlorinating Process

**DOI:** 10.1264/jsme2.ME11283

**Published:** 2011-12-06

**Authors:** Shinichi Kaiya, Sati Utsunomiya, Saori Suzuki, Naoko Yoshida, Hiroyuki Futamata, Takeshi Yamada, Akira Hiraishi

**Affiliations:** 1 Department of Environmental and Life Sciences, Toyohashi University of Technology, Toyohashi 441–8580, Japan; 2 Electronics-Inspired Interdisciplinary Research Institute, Toyohashi University of Technology, Toyohashi 441–8580, Japan; 3 Graduate School of Engineering, Shizuoka University, Hamamatsu 432–8561, Japan

**Keywords:** aromatic hydrocarbon, aromatic-ring-hydroxylating dioxygenase, dibenzofuran, polychlorinated dioxins, reductive dehalogenation

## Abstract

Aerobic aromatic-hydrocarbon-degrading bacteria from a semi-anaerobic microbial microcosm that exhibited apparent complete dechlorination of polychlorinated dibenzo-*p*-dioxins/dibenzofurans (PCDD/Fs) were isolated through enrichment and plating culture procedures with dibenzofuran as the model substrate. By 16S rRNA gene sequence comparisons, these dibenzofuran-degrading isolates were identified as being members of the phyla *Actinobacteria*, *Firmicutes*, and *Proteobacteria*, among which those of the genera *Paenibacillus* and *Rhizobium* were most abundant. All of the isolates utilized naphthalene as the sole carbon and energy source and degraded dibenzofuran metabolically or co-metabolically; however, they hardly attacked monochlorinated dibenzofuran and dibenzo-*p*-dioxin. By PCR cloning and sequencing, genes predicted to encode aromatic-ring-hydroxylating dioxygenase (AhDO) were detected in all test isolates. Real-time quantitative PCR assays with specific primer sets detected approximately 10^5^ copies of the AhDO large subunit genes g^−1^ wet wt in the microcosm from which the isolates were obtained. This order of the copy number corresponded to approximately 1% of the 16S rRNA gene copies from “*Dehalococcoides*” and its relatives present as potent dechlorinators. These results suggest that aerobic AhDO-containing bacteria co-exist and play a role in the oxidative degradation of less chlorinated and completely dechlorinated products in the PCDD/F-dechlorinating process, thereby achieving the apparent complete dechlorination of PCDD/Fs.

Biotransformation of polychlorinated dibenzo-*p*-dioxins/ dibenzofurans (PCDD/Fs) as well as of other hazardous halogenated compounds by microorganisms is well documented in connection with its implications for natural attenuation and bioremediation of organohalogen pollution (13, 20, 39, 47). One of the major modes of microbial transformation of PCDD/Fs is metabolic reductive dehaloge-nation (5, 14, 21). Some anaerobic dehalorespiring bacteria identified as “*Dehalococcoides*” and *Dehalobacter* species are able to dechlorinate selected PCDD/F congeners as terminal electron acceptors (4, 6, 12, 33, 49). Another mode of the biotransformation of dioxins and dioxin-like compounds is the oxidative degradation of their ring structures by aromatic-ring-hydroxylating dioxygenase (AhDO) having broad substrate specificity. AhDO is a multi-component enzyme system consisting of large (AhDOa) and small (AhDOb) subunits of proteins involved in the initial step of aromatic compound degradation. Large numbers of aerobic aromatic-hydrocarbon-degrading (AHD) bacteria with AhDO have been isolated and characterized from biochemical and molecular genetic points of view (13, 20, 37, 47). The available information has shown that AhDO-containing bacteria hardly attack PCDD/Fs but degrade fewer chlorinated congeners as well as dibenzo-*p*-dioxin (DD) and dibenzofuran (DF) in general.

Previously, we studied the biotransformation of PCDD/ Fs using laboratory-scale semi-anaerobic microcosms containing different levels of PCDD/Fs (22, 48). In these microcosms, all PCDD/Fs congeners were totally reduced without the significant accumulation of fewer chlorinated congeners as intermediate or end products. This was true for a fed-batch composting process loaded with high levels of PCDD/Fs (35). These microcosm and composting systems yielded high population densities of “*Dehalococcides*” and the “*Dehalococcoides*”-like group (DLG) of bacteria that might play the primary role in reductive dechlorination of PCDD/Fs. Indeed, “*Dehalococcoides*”-containing microbial consortia capable of reductive dehalogenation have been obtained from the microcosms and composting systems (15, 17, 22, 35). Our previous studies might suggest that the reductive dechlorination of PCDD/Fs and oxidative degradation of the dechlorinated products took place simultaneously, resulting in the apparent complete dechlorination of PCDD/ Fs; however, the question of whether dioxin-oxidizing and aromatic-compound-degrading bacteria actually co-exist in the PCDD/F-dechlorinating process has remained unresolved.

The present study was undertaken to isolate and characterize aerobic AHD bacteria from one of the semi-anaerobic PCDD/F-transforming microcosms we have constructed. This article reports the primary structure and phylogeny of genes encoding AhDO of the AHD isolates as well as their phenotypic traits concerning aromatic hydrocarbon degradation. Quantitative PCR (qPCR) detection of AhDOa genes specific to the isolates in the PCDD/F-dechlorinating microcosm is also reported. Ecological implications for the co-existence of aerobic AHD bacteria in the PCDD/F-dechlorinating process are discussed.

## Materials and Methods

### Microcosm and sample collection

A semi-anaerobic PCDD/F-dechlorinating microcosm designated TRS3 (22, 48), which had been constructed by seeding with river sediment and by periodic exchanges of the supernatant with OAM-2 culture medium (22), was used as the source of aerobic AHD bacteria. The sediment slurry was taken from the microcosm on days 0, 360, 460, and 570, and a portion of the sample was immediately used for the enrichment and other experiments or stored at −20°C until analysis.

### Dioxin analysis

In this study, the terms PCDD/Fs and 1-3CDD/Fs were used to denote a group of tetra- to octa-chlorinated congeners and of mono- to tri-chlorinated congeners, respectively. PCDD/Fs and 1-3CDD/Fs were extracted from the microcosm samples and analyzed by high-resolution gas chromatography-mass spectrometry (GC/ MS) as described previously (16, 22).

### Total cell counting and quinone profiling

Total bacterial counts were measured by epifluorescence microscopy with SYBR Green staining as reported previously (36). Quinones from sediment samples were extracted with an organic solvent mixture, fractionated into menaquinone and ubiquinone fractions using Sep-Pak Vac silica gel cartridges (Waters, Milford, MA, USA), and separated by reverse-phase HPLC with a photodiode array detector as described (19, 36).

### Enrichment, isolation, and cultivation

Using DF as a model substrate, AHD bacteria were enriched and isolated from the microcosm according to the previous protocol (16) with small modifications. One gram (wet wt) of the sediment slurry was suspended in 9 mL filter-sterilized phosphate-buffered saline (PBS, pH 7.0), homogenized for 1 min, and then settled for 5 min. One milliliter of the upper fraction of the homogenate was diluted serially with PBS, and these serial dilutions were used as the inoculum. Screw-capped test tubes (20 mL capacity) were used as enrichment test tubes, to which 6.5 mL DF-BSV medium (16), consisting of 6 mL vitamin-supplemented mineral medium BSV (RM2 medium [23] supplemented with vitamin solution PV1 [25]) and 0.5 mL filter-sterilized 0.2% DF solution in heptamethylnonane, were added. Alternatively, DF-containing medium was prepared by adding 100 μL of 0.1% DF solution in acetone into test tubes, evaporating on a clean bench, and then mixing with 6 mL BSV medium. The test tubes were inoculated with 100 μL diluted samples and incubated on a reciprocal shaker at 30°C for several weeks. At appropriate intervals of incubation, the enrichment cultures were streaked onto DF-overlaid 1/10 × Tryptic Soy (DFTS) agar (16) plates and incubated at 30°C for 1–3 weeks. Colonies showing a cleared “halo” formation and/or soluble yellow-pigment production were taken as being positive for degradation, and were subjected to a purification procedure by streaking agar plates. The isolates thus obtained were kept on agar slants of peptone-containing complex medium designated PBY (16) and subcultured every 3 months.

### Testing for aromatic hydrocarbon degradation

For testing the isolates for AHD activity, BSV medium (6 mL in 20-mL screw-capped test tubes) was used as the basal medium, to which biphenyl, DD, DF, 2-monochlorinated DD, 2-monochlorinated DF, or naphthalene in acetone solution as the sole carbon source was added to give a final concentration of 0.1 mM as noted above. The ability to degrade the aromatics of isolates was determined in cultures grown aerobically at 30°C by monitoring optical density at 660 nm (OD_660_), yellow metabolite production, and the concentration of a test aromatic compound remaining and salicylic acid produced as an intermediate metabolite. Aromatic compounds and salicylic acid from the cultures were extracted and measured by HPLC as described previously (25). Absorption spectra of yellow metabolites were measured with a Shimadzu Biospec-1600 spectrophotometer (Kyoto, Japan).

### Analysis of 16S rRNA gene sequences

The 16S rRNA gene from the cell lysate was PCR-amplified with a pair set of bacterial universal primers 27f and 1492r or 1525r (30) as described (18). PCR products were separated by agarose gel electrophoresis and purified using the Wizard SV Gel and PCR Clean-Up System (Promega, Madison, WI, USA) according to the manufacturer’s instructions. Purified DNA fragments were directly sequenced using a BigDye Terminator v3.1 cycle sequencing kit (Applied Biosystems, Carlsbad, CA, USA) and an Applied Biosystems 3130xl genetic analyzer. Determined sequences were assigned to the RDP taxonomic hierarchy using the CLASSIFER program (45).

### DNA extraction and purification

Bulk DNA was extracted from cells grown in PBY medium and sediment slurry samples according to the protocols previously described (22, 48). Crude DNA extracted was further purified by a standard method including deproteinization with chloroform-isoamylalcohol and RNase treatment (34). The DNA solution thus obtained was diluted in TE buffer (10 mM Tris-HCl, 1 mM EDTA, pH 8.0) as needed and used for PCR experiments.

### PCR cloning and sequencing of AhDO genes

**Fig. 1:**
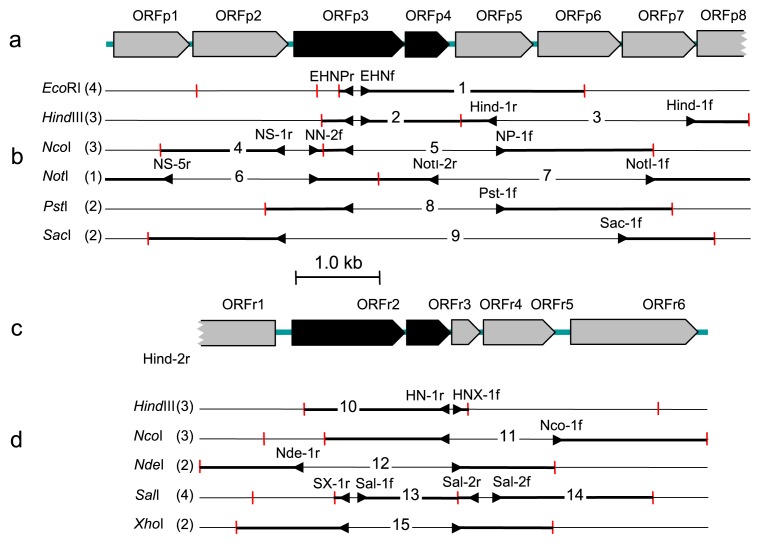
Restriction maps of and PCR cloning strategies for DNA regions containing AhDO genes from *Paenibacillus* sp. strain TSY30 and *Rhizobium* sp. strain TSY03b. (a) Arrangement of ORFs on strain TSY30 DNA, (b) restriction sites and PCR-targeted regions of strain TSY30 DNA with used primer names (see Table S1), (c) arrangement of ORFs on strain TSY03b DNA, (d) restriction sites and PCR-targeted regions of strain TSY03b DNA with used primer names (see Table S1). Large and small subunits of AhDO genes are shown by black boxes and other genes by grey boxes.

The PCR strategy and primers used in this study are summarized in Table S1 and Fig. 1. To amplify AhDOa gene fragments, degenerate PCR primers (forward primer PAH2f and reverse primers PAH3r1, PAH3r2, PAH4r, and PAH5r) were designed on the basis of AhDOa sequence information on *Burkholderia xenovorans* strain LB400^T^ (M86348), *Novosphingobium aromaticivorans* strain F199^T^ (AF079317), *Rhodococcus opacus* strain SAO101 (AB110633), *Sphingomonas wittichii* strain RW1^T^ (X72850), and *Terrabacter* (*Janibacter*) sp. strain DBF63 (AB095015). In addition, a primer set of YDA1f/YDA1r was designed to amplify the whole region of the DbfA1 gene based on sequence information on *Paenibacillus* sp. strain YK5 *dbfA1*(27). A DNA region containing an entire AhDO gene cluster was amplified by inverse PCR (38) targeting different restriction sites, for which DNA fragments produced by the digestion of genomic DNA with one of the restriction enzymes, *Eco*RI, *Hind*III, *Nco*I, *Nde*I, *Not*I, *Pst*I, *Sac*I, *Sal*I, and *Xho*I (Takara Bio, Otsu, Japan), and then ligated, were used as the template. PCR was performed using an r*Taq* DNA polymerase kit (Takara Bio), one of the primer sets, and a Takara Thermal Cycler. The first half of the PCR procedure included an initial pre-heating step of 2 min at 94°C and 20 cycles of the touch-down reaction (10) consisting of denaturation for 1 min at 94°C, annealing for 1 min at temperatures decreasing from 60 to 51°C with 1°C decremental steps of 2 cycles each, and extension for 1 min at 72°C. Following this, 20 additional cycles of the thermal reaction was performed with annealing at 50°C. The final step was followed by extension at 72°C for 2 min. PCR products were purified as noted above and subcloned using a pT7Blue-3 Perfectly Blunt^TM^ kit (Novagen, Madison, WI, USA). Transformation into *Escherichia coli* competent cells, blue/white colony selection, and plasmid extraction were performed according to the manufacturer’s instructions and standard methods of molecular cloning (41). The cloned DNA was sequenced using a cycle sequencing kit and an Applied Biosystems DNA sequencer as described above for 16S rRNA genes.

### Southern hybridization

An AhDOa gene-corresponding PCR clone amplified from each strain with a pair set of specific PCR primers (Table S1) was used as the probe for southern hybridization. For this, the probe DNA was made by labeling with digoxygenin using a DIG DNA Labeling and Detection kit (Roche Molecular Biochemicals, Indianapolis, IN, USA) according to the manufacturer’s instructions. Genomic DNA extracted and purified from the AHD isolates was digested with one of the restriction enzymes, *Eco*R1, *Not*I, *Sal*I, *Pst*I, and *Sac*I. Then, digested samples were electrophoresed on 0.8% agarose gel and blotted onto Hybond^TM^-N^+^ nylon membranes (Amersham Bioscience, Piscataway, NJ, USA) according to the standard molecular method (41). Hybridization and chemiluminescence detection of hybridized signals were performed as specified by the manufacturer.

### Phylogenetic analysis

Compilation of DNA sequence data and the conversion of the nucleotide sequences to amino acid sequences were performed using the GENETYX-MAC program (GENETYX Corporation, Tokyo, Japan). These sequence data were compared with those retrieved from the DDBJ/EMBL/GenBank databases using the BLAST homology search system (1). Multiple alignment of sequences and calculation of the nucleotide or amino acid substitution rate were performed using the CLUSTAL X ver. 2 program (31). Distance matrix trees were constructed by the neighbor-joining method (40), and the topology of the trees was evaluated by bootstrapping with 1,000 trials (11). Alignment positions with gaps were excluded from the calculations.

### Real-time qPCR detection of 16S rRNA and AhDOa genes

The bulk DNA extracted from microcosm TRS3 on days 360 and 570 was used as the template in real-time qPCR assays for the measurement of 16S rRNA and AhDOa genes. The primers used for the detection of 16S rRNA genes of total bacteria and the “*Dehalococcoides*”/DLG bacteria were a combination of 341f/534r and DHC793f/DHC946r, respectively, as described (22, 24). The specific primers used for the detection of AhDOa genes are shown in Table S1. The real-time qPCR assay was performed using a LightCycler FastStart DNA Master SYBR GREEN I kit and a LightCycler system (Roche Diagnostics, Mannheim, Germany) according to the manufacturer’s instructions and as described previously (24). The PCR procedure included pre-heating at 95°C for 10 min and 40 cycles of the reactions, each of which consisted of denaturation at 95°C for 10 s, annealing at 65°C for 4 s, and extension at 72°C for 8 s. The copy number of the amplicons was calculated using LightCycler software ver. 3.5 (Roche).

### Nucleotide sequence accession numbers

The nucleotide sequences determined in this study have been deposited under DDBJ accession numbers AB663500 to AB663506 and AB684349 for 16S rRNA genes and AB642258, AB663507, and AB684350 for AhDO gene clusters.

## Results

### Characteristics of the microcosm

The microcosm was incubated semi-anaerobically for approximately 1.6 years with periodic exchanges of the supernatant with fresh organic medium. During this period of incubation, the concentration of PCDD/Fs in the microcosm decreased to 43% on a molar basis without significant accumulation of 1-3CDD/Fs as intermediate dechlorinated products (Table 1). Congener patterns of PCDD/Fs and 1-3CDD/Fs found at the end of incubation were similar to those reported previously (22, 48) (data not shown). The microcosm yielded constant levels of total bacterial counts (7.2–8.3×10^10^ g^−1^ dry wt) and total respiratory quinones (24–30 nmol g^−1^ dry wt). The molar ratios of MK/Q were 5.1–5.6, suggesting that the microbial community in the microcosms had a tendency toward anaerobic metabolism under low-redox conditions; however, the fact that ubiquinones accounted for 18–20% of the total quinone content on average suggested that strictly aerobic and/or facultatively anaerobic bacteria with ubiquinones, *i.e.*, those of *Alpha-*, *Beta-*, and *Gammaproteobacteria*(19), constituted a significant proportion of the total microbial populations in the microcosm. The major quinone species detected as being >5 mol% on average were MK-7 (24%), MK-6 (19%), MK-8 (17%), Q-8 (8.3%), Q-10 (7.2%), MK-8(H_4_) (6.3%), and MK-8(H_2_) (5.3%).

### Isolation and identification

Aerobic AHD bacteria from microcosm TRS3 on days 0, 360, 460, and 570 were enriched using DF-BSV medium followed by streaking onto DFTS agar plates and incubating for 1 to 3 weeks. Positive colonies appearing on DFTS agar were picked up and subjected to standard purification by streaking on agar plates. We isolated 11 strains from the seed sediment (day 0) and 32 strains from the microcosm on days 360–570.

By 16S rRNA gene sequence comparisons, the AHD isolates as apparent DF degraders were classified into 11 genera and an unidentified group that belong to the phyla *Actinobacteria*, *Firmicutes*, and *Proteobacteria* (Table 2). The isolates from the microcosm on days 360–570 were taxonomically similar to each other, and those of genera *Paenibacillus* and *Rhizobium* were most abundant. Also, during this period of incubation, members of the genus *Novo-sphingobium*, possibly assignable to *N. naphthalenivorans*(44), occurred in significant numbers. This taxonomic frequency of isolation differed from that found in the microcosm on day 0, where more actinobacterial strains were isolated. Therefore, although the isolation procedure was not precisely quantitative, a population shift in AHD bacteria might take place during the start-up operation of the microcosm.

### Degradation of aromatic hydrocarbons

All of the AHD isolates were able to grow with naphthalene as the sole carbon and energy source, and the majority also utilized DF (Table 2). The *N. naphthalenivorans* and *Rhizobium* sp. isolates were unable to utilize DF as the carbon and energy source but degraded it co-metabolically in the presence of naphthalene. None of the isolates showed any or little degradation of the monochlorinated forms of DD and DF (data not shown). Chlorinated forms of DD and DF have toxic effects on microbial growth in general, while some AHD microorganisms are able to attack highly chlorinated DD and DF (13, 20, 47).

**Fig. 2:**
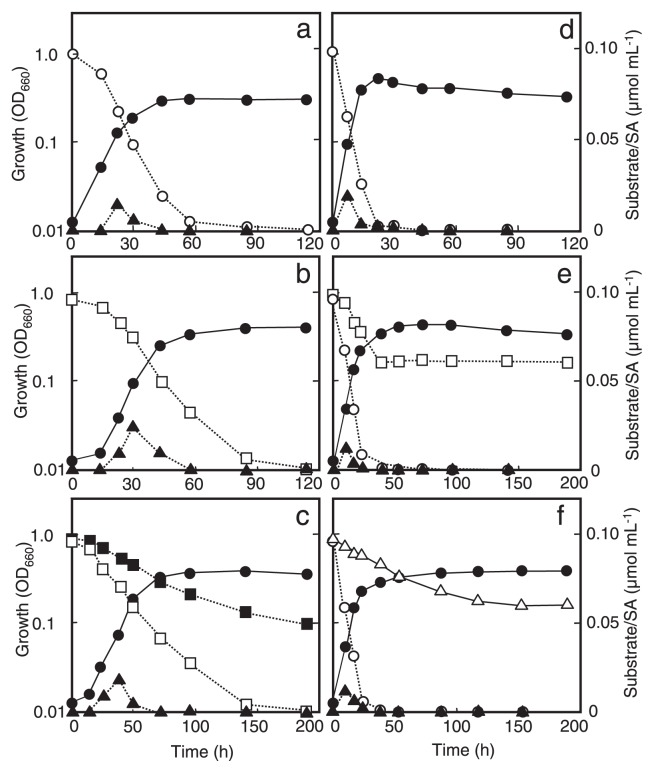
Growth of *Paenibacillus* sp. strain TSY30 and *Rhizobium* sp. strain TSY03b by degrading aromatic compounds as the substrate. (a) Degradation of naphthalene and production of salicylic acid (SA) by strain TSY30; (b) degradation of DF and production of SA by strain TSY30; (c) degradation of DD in the presence of DF by strain TSY30; (d) degradation of naphthalene and production of SA by strain TSY03b; (e) degradation of DF in the presence of naphthalene by strain TSY03b; (f) degradation of biphenyl in the presence of naphthalene by strain TSY03b. Symbols: closed circles, growth (OD_660_); open circles, concentration of naphthalene; open squares, DF; closed squares, DD; open triangles, biphenyl; closed triangles, SA.

As examples, Fig. 2 shows the growth and aromatic-compound degradation by two representatives designated as strains TSY30 and TSY03b, whose 16S rRNA gene sequences were most similar to those of *Paenibacillus naphthalenivorans* strain PR-N1^T^ and *Rhizobium selenitireducens* strain B1^T^, respectively (see Table 2). *Paenibacillus* sp. strain TSY30 degraded DF and naphthalene completely within 100 h of incubation with the production of salicylic acid as an intermediate metabolite (Fig. 2a, b). This strain attacked neither DD nor biphenyl but degraded less of the former co-metabolically in the presence of DF (Fig. 2c, f). *Rhizobium* sp. strain TSY03b consumed naphthalene completely within 24 h of incubation and degraded DF and biphenyl co-metabolically in the presence of naphthalene (Fig. 2e, f). Interestingly, the co-metabolic degradation of DF by strain TSY03b was stopped with the complete consumption of naphthalene, whereas that of biphenyl continued for a short while even if naphthalene was exhausted.

When degrading DF metabolically or co-metabolically, the majority of the isolates produced soluble yellow metabolites (Table 2), which showed a characteristic absorption maximum at a wavelength between 400 and 500 nm. There were three major patterns in the spectroscopic behavior of the yellow metabolites. Firstly, the yellow cultures of the *Mycobacterium* and *Paenibacillus* spp. isolates had an absorption maximum at 447 nm (Fig. S1a). Secondly, the yellow pigment produced by *Rhizobium* sp. isolates during the co-metabolic degradation of DF showed an absorption maximum at 417 nm (Fig. S1b). These two spectroscopic patterns remained constant independent of external pH, but the intensity of absorption increased at alkaline pH. The third pattern was obtained with the yellow pigment produced by *N. naphthalenivorans* isolates, which was characterized by a pH-dependent shift (pH 3 to 7) in the absorption maximum from 400 to 465 nm (data not shown). This is typical of 2-hydroxy-4-[3′-oxo-3′*H*-benzofuran-2′-yliden]but-2-enoic acid (HOBB), the yellow *meta*-cleavage product in the lateral dioxygenation pathway (20, 47).

### Detection of AhDOa genes

For AhDOa gene analyses, we selected three taxonomic groups of the isolates that showed different spectroscopic traits of the yellow metabolites with DF as noted above, *i.e.*, those of *N. naphthalenivorans*, *Paenibacillus* spp., and *Rhizobium* sp. PCR assays by combined use of forward primer PAH2f and reverse primer PAH3r1, PAH3r2, PAH4r, or PAH5r resulted in the successful detection of putative AhDOa gene fragments of an expected size (0.7–0.8 kb) in all test isolates (data not shown). These PCR products were subcloned, and at least 12 clones per amplicon were sequenced. As a result, the PCR clones from all *Paenibacillus* isolates produced two different sequence types (those with PAH2f/PAH3r1 [or PAH3r2] and with PAH2f/PAH5r), whereas the *N. naphthalenivorans* and *Rhizobium* sp. isolates produced one sequence type of PCR clone. Upon BLAST homology searches, translated amino acid sequences from all these DNA sequences proved to contain putative conserved domains specific to AhDOa (for details, see below). When southern hybridization with one of the PCR clones as the probe was performed against the digested genomic DNA from each strain, single positive signals were detected in all digests (Fig. S2), with few exceptions where second weaker signals were also found due to possible unspecific hybridization. This suggested that one copy of the AhDOa gene corresponding to each PCR clone is located on the genomic DNA of the AHD isolates.

Another attempt to specifically detect the DbfA1 gene fragment by PCR with a primer set of YDA1f/YDA1r also gave positive results in all *Paenibacillus* spp. isolates. This gene fragment corresponded to one of the two sequence types of the amplicons with a primer set of PAH2f/PAH5r as noted above. By subcloning and sequencing the amplicon from *Panibacillus* sp. strain TSY30, a whole DbfA1 region (1,296 bp) showing 99.8% similarity to *Paenibacillus* sp. strain YK5 *dbfA1* (AB201843) was determined.

### AhDO clusters of *Paenibacillus* and *Rhizobium* isolates

Since members of the genera *Paenibacillus* and *Rhizobium* were most frequently isolated from the microcosm as noted above (Table 2), we further determined the entire gene structure covering an AhDO gene in the two representatives, *Paenibacillus* sp. strain TSY30 and *Rhizobium* sp. strain TSY03b. In strain TSY30, at least two AhDOa genes, *i.e.*, detectable with PCR primers PAH2f/PAH3r1 and the DbfA1 gene, respectively, were present. In this study, the former gene was targeted for further study, because much less information is available on this putative gene. Our concurrent study showed that transcription of the target genes of *Paenibacillus* sp. strain TSY30 and *Rhizobium* sp. strain TSY03b as well as of the AhDOa gene of the *N. naphthalenivorans* isolates was induced by naphthalene (unpublished data). Thus, we designated these genes as naphthalene-inducible dioxygenase (NidA) genes.

A 7.3 kb nucleotide stretch of TSY30 genomic DNA that contained 8 ORF regions (designated ORFp1 to ORFp8) was identified by subcloning and sequencing the amplicons produced by inverse PCR (Fig. 1a and Table S2). As the gene homologs for large and small dioxygenase subunits are usually located in pairs, ORFp3 and the downstream region ORFp4 proved to contain the sequence corresponding to AhDOa and AhDOb, respectively. The deduced amino acid sequence of ORFp3 (NidA1) was most similar to the AhDOa sequence of *Geobacillus* sp. Y4.1MC1 (63% identity), apart from a putative AhDOa gene clone from *Paenibacillus* sp. strain YK5 (clone C89, AB201841) to which ORFp3 shows 100% identity in 83 compared residues. The deduced amino acid sequence of ORFp4 (NidA2) was closest to that of *Geobacillus* sp. Y4.1MC1 AhDOb (59% identity).

**Fig. 3:**
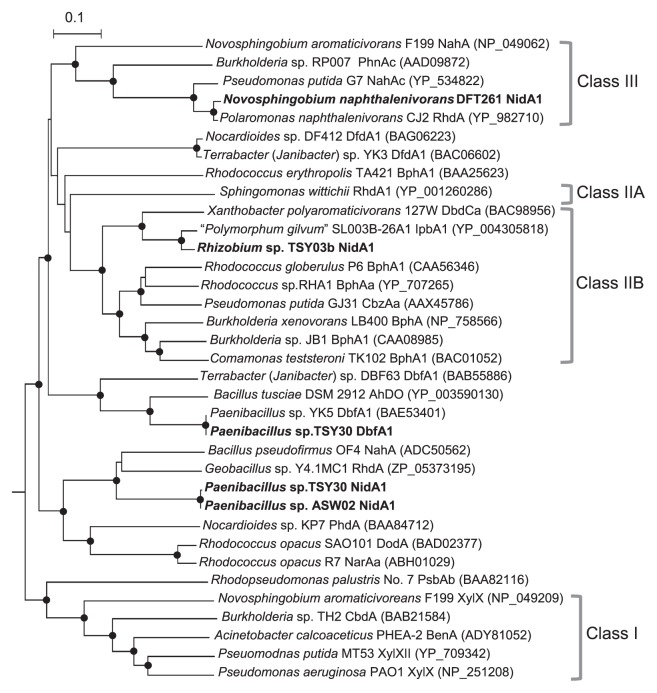
Neighbor-joining distance matrix tree of AhDOa proteins of the AHD isolates and related Rieske-type dioxygenases based on deduced amino acid sequences (scale = 10% sequence dissimilarity). The sequence of *Pseudomonas resinovorans* CA10 CarA is used as an outgroup to root the tree. The DDBJ/EMBL/GenBank accession numbers for the sequences are shown behind organism names. The sequences determined in this study are shown in bold. Branching points supported by >800 bootstrap values with 1,000 resamplings are shown by closed circles. Clusters I, II, and III show the Toluene/Benzoate, Biphenyl, and Naphthalene dioxygenase families, respectively, based on the Batie classification system (3, 42, 46).

Inverse PCR and sequencing experiments also resulted in the identification of a 5.9 kb nucleotide stretch from TSY03b genomic DNA that contained 6 ORFs (designated ORFr1 to ORFr6, Fig. 1c and Table S3). The translated amino acid sequences of ORFr2 (NidA1) and ORFr3 (NidA2) were most similar to those of the isopropylbenzene (IPB) dioxygenase large subunit (94% identity) and biphenyl 2,3-dioxygenase beta subunit (91% identity) of “*Polymorphum gilvum*” SL003B-26A1 (32), respectively. In the downstream region, two genes encoding ferredoxin (ORFr4) and dihydrodiol dehydrogenase (ORFr5) were conserved. This arrangement of catabolic genes is similar to those found in AHD bacteria containing AhDO of the class IIB dioxygenase family (see Fig. 3), such as *Pseusomonas putida* strain GJ31 (29) and *Rhodococcus globerulus* strain P6 (2).

### Phylogenetic analysis of AhDO proteins

Multiple sequence alignment analysis revealed that the Reiske-type [2Fe-2S] cluster binding site motif (Cys-X1-His-X17-Cys-X2-His) was conserved in the NidA1 and DbfA1 of *Paenibacillus* sp. strain TSY30 and the NidA1 of *Rhizobium* sp. strain TSY03b. The corresponding His residue (-Cys-X2-His) in AhDO proteins was found in all other test isolates including *N. naphthalenivorans* strain DFT261. We also found the conserved mononuclear nonheme Fe(II) active site coordinated by His-214, His-219, and Asp-370 in the TSY30 NidA1 and by His-220, His-226, and Asp-371 in the TSY03b NidA1.

A neighbor-joining phylogenetic tree based on the translated amino acid sequences from AhDOa genes revealed that there was a great diversity in putative AhDOa proteins among the AHD isolates (Fig. 3). The NidA1 proteins of *Rhizobium* sp. strain TSY03b and *N. naphthalenivorans* strain DFT261 were positioned in the Biphenyl (class IIB) and Naphthalene (class III) dioxygenase families, respectively, based on the Batie classification system (3, 42, 46). On the other hand, the NidA1 and DbfA1 of *Paenibacillus* sp. strains TSY30 and ASW02 formed respective distinct lineages not categorized by this classical system of grouping.

### qPCR detection of AhDO genes in microcosm

While the AHD isolates we obtained were strictly aerobic, the PCDD/F-transforming microcosm from which they were isolated had been continuously kept under (semi-)anaerobic conditions; therefore, it is of special interest to check the population density of AHD bacteria in the microcosm to elucidate their ecological significance. Real-time qPCR assays targeting AhDOa genes showed that each of the AhDOa genes we tested occurred in the order of 10^4^–10^5^ copies g^−1^ wet wt (Table 3). On the other hand, the 16S rRNA genes of total bacteria and “*Dehalococcoides*”/DLG bacteria detected were 1.7–3.3×10^10^ and 1.4–8.7×10^7^ copies g^−1^ wet wt, respectively. These data might suggest that the population density of the co-existing AhDO-containing bacteria as estimated by the sum of the test gene copies (1.4–1.6×10^5^ copies g^−1^) account at least for 1% of those of the dehalorespiring bacteria in the microcosm.

## Discussion

We have observed that apparent complete dechlorination of PCDD/Fs takes place in the semi-anaerobic microcosm under given conditions, as reported here and previously (22, 48). Our assumption to explain this observation was that aerobic and facultatively anaerobic bacteria co-existed and played major roles in the oxidative degradation of fewer chlorinated and completely dechlorinated products in the microcosm. Previous clone library analyses targeting 16S rRNA genes have shown that possibly strict anaerobes belonging to *Bacteroidetes*, *Firmicutes*, and *Deltaproteo-bacteria* predominate in this semi-anaerobic process (22, 48). The high MK/Q ratio found in the microcosm in this study showed its low redox state to allow the proliferation of anaerobic bacteria, but also suggested the occurrence of ubiquinone-containing aerobic proteobacteria in a significant proportion. In this study, we successfully isolated a number of aerobic AHD bacteria from the microcosm at different stages and characterized their AhDO genes possibly involved, thereby confirming the co-existence and potential ecological roles of aerobic AHD bacteria in the PCDD/F-transforming microbial community.

The results of 16S rRNA gene sequencing of the AHD isolates showed that they were phylogenetically diverse, among which members of the genera *Paenibacillus* and *Rhizobium* were most abundant. Although why these taxonomic groups of bacteria were most frequently isolated from the microcosm is not known, this finding contrasts with the observation that actinobacterial DF-degrading bacteria were more abundant in the sediment used as the seed for the microcosm (see Table 2) and in PCDD/F-contaminated environments previously studied (16, 28). Most of our isolates could be assigned to members of the established genera belonging to the phyla *Actinobacteria*, *Firmicutes*, and *Proteobacteria*, showing the high similarities of 16S rRNA gene sequences to previously established species at >98% similarity levels. For example, *Paenibacillus* sp. strain TSY30 as a representative from one of the major taxonomic groups occurring abundantly is closely related to the type strain of *Paenibacillus naphthalenovorans* (99.2% similarity), which was created to accommodate a group of AHD bacteria isolated from the rhizosphere of salt marsh plants (8, 9). Similarily, *Rhizobium* sp. strain TSY03b, a member of another abundant taxonomic group, is phylogenetically most related to the type strain of *Rhizobium selenitireducens* (99.0% similarity), a selenate-reducing bacterium not known to have AHD activity (26). The above-noted levels of 16S rRNA gene sequence similarity between the isolates and their closest relatives are close to the threshold of the similarity level (98.7–99.0%) shown by a single species (43). Genomic DNA-DNA hybridization studies as well as more phenotypic characterization should give definite information about the exact taxonomic position of the AHD isolates at the species level.

There are two major modes of initial oxidation of DD and DF catalyzed by AhDO (20, 47). One is termed lateral dioxygenation, which is characterized by attacks on the aromatic rings usually at the lateral 1,2 positions and by production of the yellow metabolite, HOBB. The other is angular dioxygenation, taking place at the angular positions 4 and 4a adjacent to the ether bridge (37). In the case of the *N. naphthalenivorans* isolates, it is likely that lateral dioxygenation is involved in the co-metabolic degradation of DF, in view of their production of a yellow metabolite possibly as HOBB and the detected putative gene encoding AhDOa of the Naphthalene dioxygenase family. On the other hand, the *Paenibacillus* and *Rhizobium* isolates produced the respective yellow metabolites from DF that differ from HOBB in spectroscopic behavior. Functional gene analyses have demonstrated that *Paenibacillus* sp. strain TSY30 has at least two putative AhDO genes encoding NidA1 and DbfA1 proteins, the latter of which is closely related to *Paenibacillus* sp. YK5 DbfA1, being responsible for angular dioxygenation of DF (27). In the case of strain TSY30, therefore, DF might be degraded with the concomitant production of the yellow metabolite via an angular dioxygenation pathway catalyzed by DbfA1, although no information is available on whether *Paenibacillus* sp. YK5 produces the yellow metabolite. The putative AhDO gene of *Rhizobium* sp. strain TSY03b is closely related to that of “*Polymorphum gilvum*” (32), an alphaproteobacterium isolated from crude oil-contaminated saline soil (7), and this gene may be involved in its co-metabolic degradation of DF. Further study should elucidate the metabolic pathway for the degradation of DF and related aromatic compound and the identification of the yellow metabolite in *Paenibacillus* and *Rhizobium* isolates.

Phylogenetic analysis based on the amino acid sequences deduced from the putative AhDO genes of the AHD isolates in comparison with those of previously known Rieske-type dioxygenases have shown a great diversity of AhDO proteins found in the PCDD/F-transforming microbial community. The NidA1 of *Paenibacillus* isolates belongs to an AhDOa cluster deeply branching off from previously known AhDOa families, including those defined by the Batie classification system (3, 42, 46). This suggests that TSY30 *nidA1* is a novel type of gene possibly involved in naphthalene degradation. Moreover, it is of special interest that the NidA1 of *Rhizobium* sp. strain TSY03b belongs to the Biphenyl dioxygenase family despite its specificity to naphthalene rather than biphenyl as the substrate (unpublished data, also see Fig. 2f). Therefore, it is difficult to predict the function and specificity of AhDO proteins from phylogenetic data as well as from information on the primary structure of the encoding genes. Further study is clearly necessary to fully elucidate the functional role of NidA genes in AHD isolates.

The semi-anaerobic microcosm as the origin of AHD isolates was capable of reductive dechlorination of PCDD/ Fs, for which “*Dehalococcoides*”/DLG bacteria were probably responsible, as described previously (22, 48). Real-time qPCR assays showed that “*Dehalococcoides*”/DLG bacteria occurred in 10^7^ cells g^−1^ in this microcosm, suggesting the high activity of reductive dechlorination. As reported herein and previously (22, 48), however, 1-3CDD/Fs and the dechlorinated products (*i.e.*, DD and DF) in the microcosm occurred only at pmol (per g) levels that were much lower than those predicted on the basis of the dechlorination rate recorded for the microcosm. In view of the amount of the less chlorinated and dechlorinated products actually found in the microcosm, the availability of these compounds as carbon and energy sources for aerobic AHD bacteria might not be high. The sum of the tested AhDOa gene copies detected in the microcosm (10^5^ copies g^−1^ wet wt) suggests that the growth of aerobic AHD bacteria was actually limited by such low availability of the substrate as well as of dissolved oxygen required for its oxidation. The microcosm was supplied with dissolved oxygen via periodic medium exchange, which might occur in trace amounts therein but be actually enough to support the growth of aerobic AHD bacteria with small amounts of the substrate.

In summary, aerobic AhDO-containing bacteria co-exist and may play an important role in the oxidative degradation of fewer chlorinated and completely dechlorinated products and their metabolites in the PCDD/F-transforming process, thereby achieving the apparent complete dechlorination of PCDD/Fs. Our concurrent study has shown that sediment from the microcosm rapidly degrades DF and naphthalene after some lag when exposed to aerobic conditions (unpublished data). Since we used DF only for the initial isolation of AHD bacteria from the microcosm, it can be presumed that much more phylogenetically and functionally diverse AHD bacteria than we could find are present in this process. In this study, our attempt to isolate bacteria capable of degrading 1-3CDD/Fs produced negative results, but it may be logical to predict the occurrence of such chlorinated-dioxin-oxidizing bacteria in the PCDD/F-transforming microbial community.

## Supplementary material



## Figures and Tables

**Table 1:** Dioxin concentrations, direct total bacterial count, and respiratory quinone content in the test microcosm^a^

Day of sampling	PCDD/Fs (pmol g^−1^dry wt)	PCDD/Fs (pg-TEQ g^−1^dry wt)	1-3CDD/Fs (pmol g^−1^ dry wt)	Total count (cells×10^10^ g^−1^ dry wt)	Quinone content (nmol g^−1^ dry wt)	

					Q	MK
0	2,800±140	1,700±140	91±7	0.034	NT^b^	NT
360	1,500±120	910±90	87±8	8.3±1.7	5.4±0.9	30.0±1.5
460	1,200±110	660±65	68±9	7.9±1.8	NT	NT
570	950±110	570±33	46±3	7.2±1.1	5.1±0.7	26.0±1.4

a Data shows the average ± standard deviation (*n*=3).

b NT, not tested.

**Table 2:** Phylogenetic identification of AHD isolates from the microcosm and their ability to utilize biphenyl (BP), dibenzo-*p*-dioxin (DD), and naphthalene (NP) in addition to dibenzofuran (DF) as the sole carbon and energy source

RDP taxonomic hierarchy assignment	16S rRNA gene comparison		No. of strains isolated^a^	Utilization of:^b^			
	
	Closest relative as the type strain (accession number)	Similarity (%)		BP	DD	DF	NP
*Actinobacteria*							
*Janibacter*	*J. terrae* CS12^T^ (AF176948)	99.8	1 (1, 0, 0, 0)	nt	nt	+	+
*Mycobacterium*	*M. moriokaense* DSM 44221^T^ (AJ429044)	98.9–99.2	3 (0, 2, 1, 0)	+y	−	+y	+
*Nocardioides*	*N. aromaticivorans* H-1^T^ (AB087721)	99.9–100	2 (2, 0, 0, 0)	−	−	+y	+
*Rhodococcus*	*R. qingshengii* djl-6^T^ (DQ090961)	99.2–100	4 (4, 0, 0, 0)	+y	−	+y	+
*Sinomonas*	*S. atrocyaneus* DSM 20127^T^ (X80746)	99.6	2 (1, 1, 0, 0)	−	−	+	+
*Firmicutes*							
*Paenibacillus*	*P. naphthalenovorans* PR-N1^T^ (AF353681)	99.1–99.2	9 (1, 3, 2, 3)	−	−^+1^	+y	+
	*P. borealis* KK19^T^ (AJ011322)	97.7	2 (0, 1, 0, 1)	−	−^+1^	+	+
*Proteobacteria*							
*Novosphingobium*	*N. naphtalenivorans* TUT562^T^ (AB177883)	99.9–100	6 (1, 2, 2, 1)	−	−	−^+2^y	+
*Rhizobium*	*R. selenitireducens* B1^T^ (EF440185)	98.9–99.0	9 (0, 4, 2, 3)	−^+2^y	−^+2^	−^+2^y	+
*Sphingomonas*	*S. wittichii* RW1^T^ (AB021492)	99.9	1 (1, 0. 0, 0)	nt	nt	+	+
*Variovorax*	*V. paradoxus* DSM 66^T^ (AJ420329)	99.6	1 (0, 1, 0, 0)	nt	nt	+y	+
*Dokdonella*	*D. koreensis* DS-123^T^ (AY987368)	95.4–95.5	1 (0, 0, 1, 0)	nt	nt	+y	+
Unidentified	*H. effusa* AP103^T^ (AY363245)	94.7–95.0	2 (0, 0, 2, 0)	nt	nt	+y	+

a Figures in parentheses show the number of strains isolated from the microcosm on days 0, 360, 460, and 570 in order.

b Abbreviations and symbols: +, utilization positive; +y, utilization and yellow metabolite production positive; −, utilization negative, −^+1^, co-metabolic degradation positive with DF; −^+2^, co-metabolic degradation positive with naphthalene.

**Table 3:** Real-time qPCR-estimated copy numbers of AhDOa genes of AHD bacteria in the microcosm

Target AhDO gene from:	Abundance of AhDOa genes (copies g^−1^) on days:	

	360	570
*Paenibacillus* sp. TSY30 (NidA1)	1.4±0.5×10^5^	5.0±0.6×10^4^
*Paenibacillus* sp. TSY30 (DbfA1)	1.0±0.5×10^5^	4.3±0.4×10^4^
*Novosphingobium naphthalenivorans*	3.2±0.6×10^4^	4.5±0.5×10^4^
DFT261		
*Rhizobium* sp. TSY03b	1.1±0.4×10^4^	4.8±0.5×10^4^

## Non-standard abbreviations

AHD,: aromatic hydrocarbon degrading
AhDO,: aromatic-ring-hydroxylatingdioxygenase
AhDOa,: aromatic-ring-hydroxylating dioxygenase large subunit
AhDOb,: aromatic-ring-hydroxylating dioxygenase small subunit
DbfA1,: dibenzofuran dioxygenase large subunit
DD,: dibenzo-*p*-dioxin
DF,: dibenzofuran
HOBB,: 2-hydroxy-4-[3′-oxo-3′*H*-benzofuran-2′-yliden]but-2-enoic acid
NidA1,: naphthalene-inducible dioxygenase large subunit
NidA2,: naphthalene-inducible dioxygenase small subunit
PCDD/ Fs,: polychlorinated dibenzo-*p*-dioxins/dibenzofurans.
