# Supplementary material for: Isolation and Functional Gene Analyses of Aromatic-Hydrocarbon-Degrading Bacteria from a Polychlorinated-Dioxin-Dechlorinating Process
Source: Microbes Environ. 2011 Dec 6;27(2):127–35. doi: 10.1264/jsme2.ME11283 (PMC4036015; doi:10.1264/jsme2.ME11283)

Supplemental tables

Table S1. PCR primers used for molecular analyses of DF-degrading bacteria

PCR strategy and target regions	Primer name	Direction	Sequence (5' - 3')
Standard PCR			
AhDOa	PAH2f	Forward	TCVBSTGCMVYTAYCAYGGYTGG
	PAH3r1	Reverse	TCAYCYGKTCCYYTMGGRTGCCA
	PAH3r2	Reverse	TYATBSGGMCCVMKCGGVTGCCA
	PAH4r	Reverse	CSATSRMATASKTCCAVACYTCG
	PAH5r	Reverse	CCRTCRTCSKGBKCRAAHAMACC
Paenibacillus DbfA1	YDA1f	Forward	GGAGCAGCATGTAGTCAGAG
	YDA1r	Reverse	GATCCAGCAAGTGAGCTTCG
Inverse PCR for *Paenibacillus* sp. TSY30			
1, 2, 3*	EHNf	Forward	AGTCCCAAGTCCAAAGCCAGAC
1, 2, 3, 5	EHNPr	Reverse	CTTCCAGTTCGCATGAACGACC
4, 8	NN-2f	Forward	ATGATCCGGTACTGTTGGTGCG
4	NS-1r	Reverse	TCCTGCTGGTAGGCCATAAACG
5	Pst-1f	Forward	TCTGATCATTGCGGTGTCGCTG
6	SacI-1f	Forward	AGCCTTGATGGAGGACTACGAG
6, 8	SN-5r	Reverse	GCAGCAATCCCAGATGATCTCG
7	Not-1f	Forward	GTTCAAGGATACGGATCTGGCC
7	Not-2r	Reverse	GTGACTGCGAACGCTTAGTTCC
Inverse PCR for *Rhizobium* sp. TSY03b			

10, 12, 15*	HNX1f	Forward	ATGATGATCTACCGCACGCGGC
10, 11	HN-1r	Reverse	ACCATAGGTGTACGCGAAGC
11	Nco-1f	Forward	GACTGACAACGAAACCTTCGCC
12	Nde-1r	Reverse	TTGGCAATCTGCGACTCATGGC
13	Sal-1f	Forward	GAATCTGTCGTTCCTGGCAG
13, 15	SX-1r	Reverse	GATGACCCACTTCTGGATGC
14	Sal-2f	Forward	GATACGTGGAAGAGGGTGCAC
14	Sal-2r	Reverse	CAGCTTGCAGATCAGGATCGG
Real-time qPCR for AhDOa from:			
Paenibacillus sp. (NidA1)	TSY30n-2f	Forward	TGGGTCGTTCATGCGAACTG
	TSY30n-2r	Reverse	CCACATTTCTTGCGGCAACC
Paenibacillus sp. (DbfA1)	TSY30d-1f	Forward	TTGAAGTGGCAGGTCCCATC
	TSY30d-1r	Reverse	CGATCCAATTCCAGACCCAC
Rhizobium sp. TSY03b	TSY03b-1f	Forward	GCACGACCTCACATCTGTCCGGCATC
	TSY03b-1r	Reverse	GACACAAGTGTACATCGGTCCGCGAC
N. naphthalenivorans DF261	DF261-1f	Forward	GGAGCCCATCTTCAAGCACTCA
	DF261-1r	Reverse	CCGGAATAAGCGTCCCACAACA

* Figures correspond to the DNA fragment regions shown in Fig. 1b and 1d.

Table S2. Deduced ORFs found in the 7.3 kb nucleotide stretch of cloned DNA from *Paenibacillus* sp. strain TSY30

ORF	Position in accession no.	Calculated molecular mass (Da)	BLAST homology search for protein			
			% Identity	Protein/function	Closest relative as host organism	Accession number
1	56–925	32,812	65	5-Carboxymethyl-2-hydroxymuconate delta-isomerase	*Bacillus tusciae* DSM 2912	YP_003590136
2	944–2137	44,718	55	Monooxygenase FAD-binding protein	*Geobacillus* sp. Y412MC61	ZP_04392202
3	2205–3533	50,860	63	Aromatic-ring-hydroxylating dioxygenase, alpha subunit	*Geobacillus* sp. Y4.1MC1	ZP_05373195
4	3552–4049	19,431	59	Aromatic-ring-hydroxylating dioxygenase, beta subunit	*Geobacillus* sp. Y4.1MC1	ZP_05373196
5	4106–4987	31,337	40	Inner-membrane translocator	*Geobacter lovleyi* SZ	YP_001952098
6	5027–6022	35,919	41	Inner-membrane translocator	*Geobacter uraniumreducens* Rf4	ZP_01141122
7	6019–6810	28,905	50	ABC transporter-related ATP-binding protein	*Acidiphilium cryptum* JF-5	YP_001233501
8	6798-7348*	–	56	ABC transporter	*Paenibacillus* sp. D14	ZP_04853801

* Incomplete sequence in the 3' region.

Table S3. Deduced ORFs found in the 5.9 kb nucleotide stretch of cloned DNA from *Rhizobium* sp. strain TSY03b

ORF	Position in accession no.	Calculated molecular mass (Da)	BLAST homology search for protein			
			% Identity	Protein/function	Closest relative as host organism	Accession number
1	898–1*	–	89	TRAP transporter solute receptor, TAXI family	*Polymorphum gilvum* SL003B-26A1	YP_004305816
2	1106–2466	50,644	94	IPB-dioxygenase, ISP large subunit	*Polymorphum gilvum* SL003B-26A1	YP_004305818
3	2536–3111	22,832	91	Biphenyl 2,3-dioxygenase beta subunit	*Polymorphum gilvum* SL003B-26A1	YP_004305819
4	3122–3427	11,007	71	Ferredoxin	*Xanthobacter polyaromaticivorans*	BAC98958
5	3461–4294	29,127	89	Short-chain dehydrogenase/reductase (dihydrodiol dehydrogenase)	*Polymorphum gilvum* SL003B-26A1	YP_004305821
6	4450–5901	51,690	90	Dehydrogenase PhnF	*Polymorphum gilvum* SL003B-26A1	YP_004305822

*Incomplete sequence in the complementary 3' region.

Supplemental figures

Fig. S1. Absorption spectra of the yellow-pigmented DF-degrading cultures of *Paenibacillus* sp. strain TSY30 (a) and *Rhizobium* sp. strain TSY03b (b). DF in the culture of *Rhizobium* sp. strain TSY03b was degraded co-metabolically in the presence of naphthalene. Spectral patterns of the pigmented supernatant in response to changes in pH are shown.

Fig. S2. Southern hybridization of digested genomic DNA of *Paenibacillus* sp. strain TSY30 (a, b) and *Rhizobium* sp. strain TSY03b (c, d) with a labeled PCR clone as the probe. The probes used were the PCR clone with primers PAH2f/PHA3r2 (*nidA1*) for *Paenibacillus* sp. strain TSY30 and that with primers PAH2f/PHA5r (*nidA1*) for *Rhizobium* sp. strain TSY03b. (a, c), Agarose electrophoresis with ethidium bromide staining of genomic DNA digested with restriction enzymes, *Eco*RI, *Not*I, *Sal*I, *Pst*I, and *Sac*I; (b, d), hybridization signals on the nylon membrane to which the digested DNAs were transferred. Positive controls are indicated in lane P.

Kaiya et al. Fig. S1

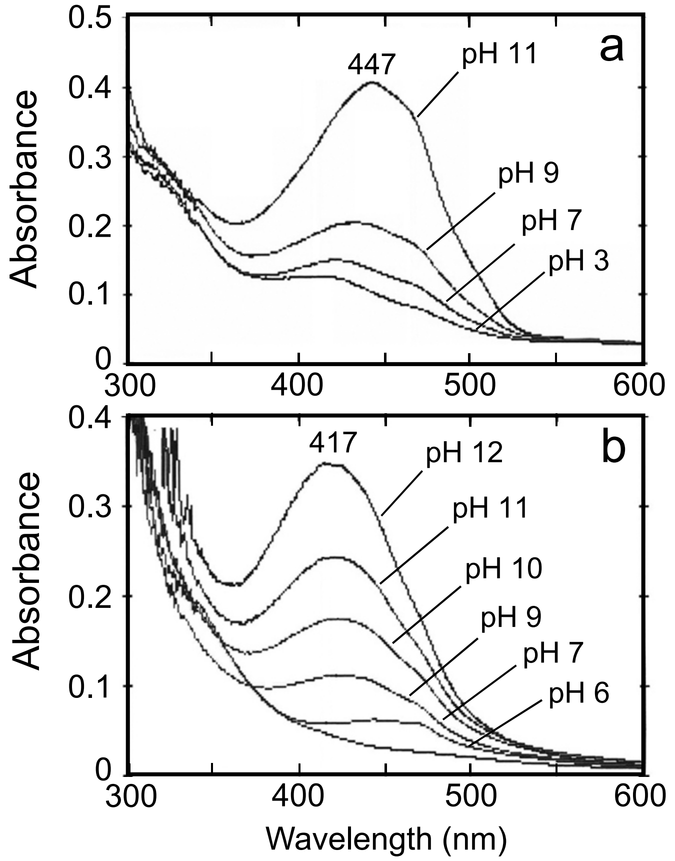

Kaiya et al. Fig. S2

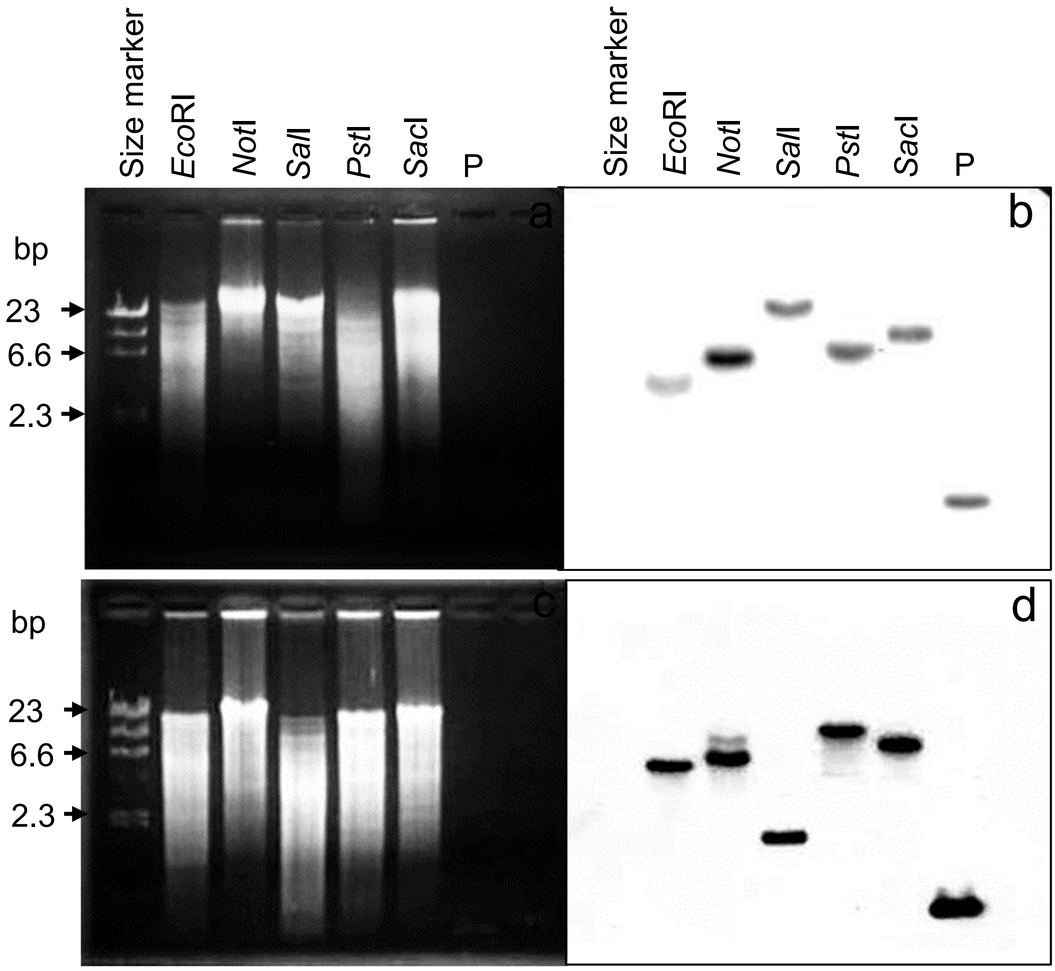

Supplement: Supplementary file 1 [file 27_127_s1.pdf]
